# Supplementary material for: The Native Ant Lasius niger Can Limit the Access to Resources of the Invasive Argentine Ant
Source: Animals (Basel). 2020 Dec 21;10(12):2451. doi: 10.3390/ani10122451 (PMC7767331; doi:10.3390/ani10122451)
Supplement: Supplementary file 1 [file animals-10-02451-s001.pdf]

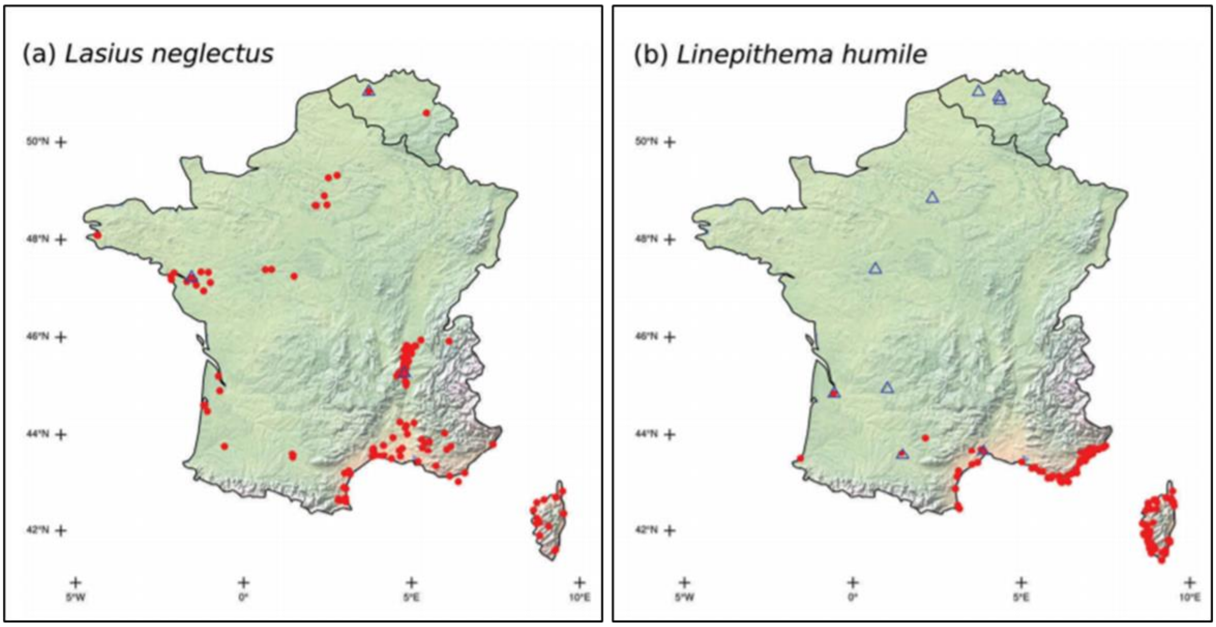

Figure S1. Occurrences of the introduced species *Lasius neglectus* (a) and *Linepithema humile* (b) in the studied area (from [1]). Outdoors locations are indicated by red dots and indoors locations by blue triangles. *Lasius niger* is present in the whole studied area [2].

1. Blatrix, R.; Colin, T.; Wegnez, P.; Galkowski, C.; Geniez, P. Introduced ants (Hymenoptera: Formicidae) of mainland France and Belgium, with a focus on greenhouses. *Ann Soc Entomol Fr* **2018**, 54, 293-308.
2. Lebas, C.; Galkowski, C.; Blatrix, R.; Wegnez, P. Fourmis d'Europe occidentale. **2016**. Delachaux et Niestlé, Paris.

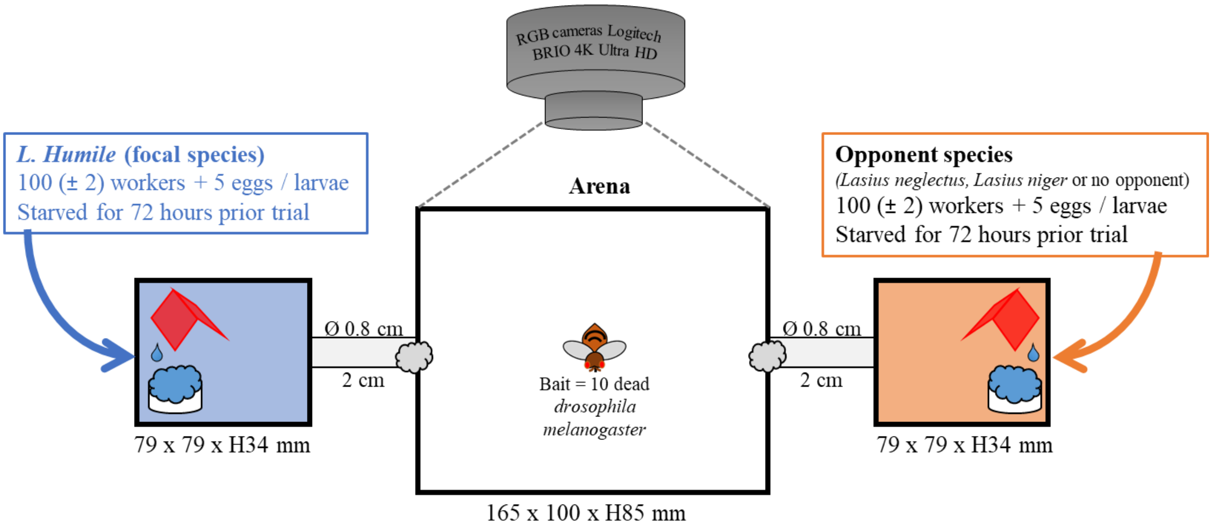

Figure S2. Schema of the experimental design used for the interactions study. Two small peripheral plastic boxes have been connected by small plastic tube to a central arena. These boxes were characterized by an area of resting site opposite the arena, with a source of humidity and an area covered with a red filter (favorable brightness). One hundred (± 2) workers were placed in each peripheral box with 5 brood elements. The ants were starved for 72 hours prior to each trial. One of the connections between the peripheral boxes and the arena was opened during 72 h before the test (Resident) while the other was closed until the start of the test (Colonizer).
